# Enhanced neuroplasticity and gait recovery in stroke patients: a comparative analysis of active and passive robotic training modes

**DOI:** 10.1186/s12883-025-04226-0

**Published:** 2025-05-31

**Authors:** Yong Yu, Wenhao Huang, Halikejiang Tuerxun, Yadan Zheng, Liujie Su, Xin Li, Zulin Dou

**Affiliations:** 1 Department of Rehabilitation Medicine, The Third Affiliated Hospital of Sun Yat- Sen University, Guangzhou, China; 2 Department of Rehabilitation Medicine, The First People’s Hospital of Kashi, Kashi, China

**Keywords:** Stroke, Gait recovery, Robotic training, Active mode, Passive mode, Motor function, Neurophysiological assessment, Rehabilitation

## Abstract

**Background:**

Stroke is a leading cause of long-term disability, with lower limb dysfunction being a common sequela that significantly impacts patients' mobility and quality of life. Robotic-assisted training has emerged as a promising intervention for gait rehabilitation post-stroke. This study aims to compare the effects of active and passive lower limb robotic training on gait recovery in stroke patients.

**Methods:**

This randomized controlled trial included 45 stroke patients who were divided into three groups: active mode group, passive mode group, and control group. All participants received standard rehabilitation therapy, while the intervention groups additionally received 20 min of robotic training (active or passive mode) daily for 10 sessions over two weeks. Outcome measures included the Fugl-Meyer Assessment (FMA) for motor function, motor evoked potentials (MEP) for neurophysiological assessment, and functional near-infrared spectroscopy (fNIRS) for brain imaging.

**Results:**

Both active and passive groups showed significant improvements in FMA scores and MEP measures compared to pre-treatment baselines (*P* < 0.01). The active group exhibited significantly greater FMA score improvements (*P* = 0.02) and MEP amplitudes (*P* < 0.01) than the passive group. Additionally, fNIRS results indicated significantly enhanced brain activation in the affected motor cortex in the active group post-treatment (*F* = 5.82, *P* = 0.026), a change not observed in the passive group. These findings underscore the clinical superiority of active robotic training in enhancing motor recovery post-stroke.

**Conclusion:**

Active mode robotic training is more effective than passive mode training in improving motor function and neurophysiological outcomes in stroke patients. These findings support the preferential use of active mode robotic training in clinical rehabilitation settings for enhancing gait recovery post-stroke. Further research with larger sample sizes and longer follow-up periods is warranted to confirm these results and explore long-term benefits.

## Introduction

Stroke remains a prevalent central nervous system disorder, with approximately 15 million new cases globally each year, one-third of which result in varying degrees of functional impairment, significantly impacting quality of life and societal participation [1, 2]. Lower limb motor dysfunction is one of the common sequelae after stroke, which is manifested as unsteady gait and difficulty in walking. Restoring lower limb function and improving walking ability is one of the important goals of stroke rehabilitation [3].

In recent years, robot-assisted rehabilitation training has been gradually applied to the clinic and has shown better results [4, 5]. Lower limb robots can effectively promote the recovery of neurological function in stroke patients by providing repetitive, high-intensity and controlled exercise training [5]. Depending on the exercise mode provided by the lower limb robot, it can be categorized into active assisted and passive modes [6, 7]. The active assisted mode helps patients to complete autonomous movement by providing partial assistive force through the robot, and is suitable for patients with partial muscle strength recovery; while the passive mode completely replaces the patient's movement through the robot, and is suitable for patients with extremely weak muscle strength or complete loss of the ability of autonomous movement.

While numerous studies have established the efficacy of lower limb robotic training in enhancing walking function in stroke patients, the differential impact of active assisted versus passive training modalities on functional recovery remains underexplored [8–10]. For example, Husemann et al. (2007) found in a randomized controlled trial that gait training using a lower-limb robot significantly improved walking speed and step length in stroke patients [9, 11]. However, how differences in training modes affect rehabilitation outcomes remains an unanswered question. Recent studies have begun to address these gaps by comparing various robot-assisted training modes, revealing differential effects on motor recovery and functional outcomes [12–14].

Active assisted and passive modes are the two main modes of lower limb robotic training. The active assisted mode helps the patient to accomplish autonomous movements by providing partial assistive force through the robot, which is believed to promote neuroplasticity and recovery of autonomous motor functions [15, 16]. For example, Hidler et al. (2009) found that active assisted mode training improved patients'walking speed and gait symmetry[17, 18]. In contrast, the passive mode completely replaces the patient's movement by the robot and is primarily used for patients with extremely weak muscle strength or complete loss of voluntary movement [19, 20]. Although the passive mode can provide highly repetitive and consistent training, its effect on neurological recovery may not be as significant as the active assisted mode [6, 21].

Therefore, this study aimed to compare the effects of lower limb robotic training in active assisted and passive modes on the recovery of walking function in stroke patients, and to explore its application value in rehabilitation. The rehabilitation effects of different training modes were analyzed by evaluating the motor function, neurophysiological indexes and functional brain imaging of each group of patients before and after training, with the aim of providing more evidence-based basis for stroke rehabilitation.

## Methods

### Participants selection

In accordance with the Declaration of Helsinki, This is a single-center study, which was approved by the ethics committee of the Third Affiliated Hospital of Sun Yat-sen University (Ethics No. [2021]02–333-01) and registered under the trial identifier ChiCTR2100054527 on the Chinese Clinical Trials Registry website (Registration date: December 19, 2021). Stroke patients hospitalized in the Rehabilitation Department of the hospital between September 2021 and May 2024 were included. The enrolled patients signed a written informed consent after clearly understanding the purpose and process of the study [22, 23].

### Inclusion and exclusion criteria

Inclusion criteria: 1) age between 25 and 80 years old, first onset of the disease, and duration of the disease from 3 weeks to 6 months; 2) diagnosis of unilateral stroke, confirmed by cranial CT or MRI [24]; 3) presence of walking dysfunction, with a sitting balance of grade 1 or higher [25]; and 4) the patient or his/her family members had signed an informed consent form [26].

Exclusion criteria: 1) history of craniocerebral trauma or other neurological disorders; 2) those with pacemakers, intracranial metal implants, or cranial defects; 3) the presence of severe cognitive or communication disorders [27]; 4) a history of epilepsy or being in the middle of pregnancy; and 5) major organ dysfunction or other serious physical diseases [28, 29].

### Sample size estimates and grouping

The sample size was calculated using G*Power 3.1, with assumptions of α = 0.05, test power (1-β) of 0.80, and an effect size of 0.5. Considering a potential 20% dropout rate, a total of 45 patients were recruited to ensure sufficient power for detecting significant differences among groups [17]. Patients were randomized into three groups of 15: Active-assisted therapy group, Passive therapy group and control group. Participants were randomly assigned to three groups using a pre-generated random number table. The allocation was performed by an independent researcher, and the sequence was concealed in sealed, opaque envelopes to ensure allocation concealment. Both outcome assessors and data analysts remained blinded to group assignments to minimize bias [30, 31].

### Interventions

In this study, the Active-assisted therapy group was trained using the lower limb robot assisted mode for 10 training sessions lasting 20 min each, once a day, 5 days a week [32, 33]. Besides, the Active-assisted gait training was conducted using A lower limb robot, developed by Beijing Ai-Robotics Technology Co., Ltd., known as AI-ROBOTICS (www.ai-robotics.cn), with the following parameters: 30–40 degrees of Hip flexion angle, 20–30 degrees of Knee flexion angle, approximately 0.4–0.8 m/s of Gait speed (the speed to match the patient's comfortable walking speed). Assistance was calibrated to provide partial assistance (50–70%) based on the patient's need for movement assistance. These settings were chosen based on recommendations in the literature that found these parameters to be effective in improving gait function in stroke patients [17, 34].

The Passive therapy group, on the other hand, performed the same training conditions using the passive mode of the lower limb robot for 20 min per session, once a day, 5 days a week, for a total of 10 sessions [7]. Both groups also received 30 min of regular rehabilitation including trunk core muscle control, balance training, transfer training, and walking training. The control group received only the same routine rehabilitation as the other two groups, but without the use of the lower limb robot [35].

### Outcome measurements

**Fig. 1:**
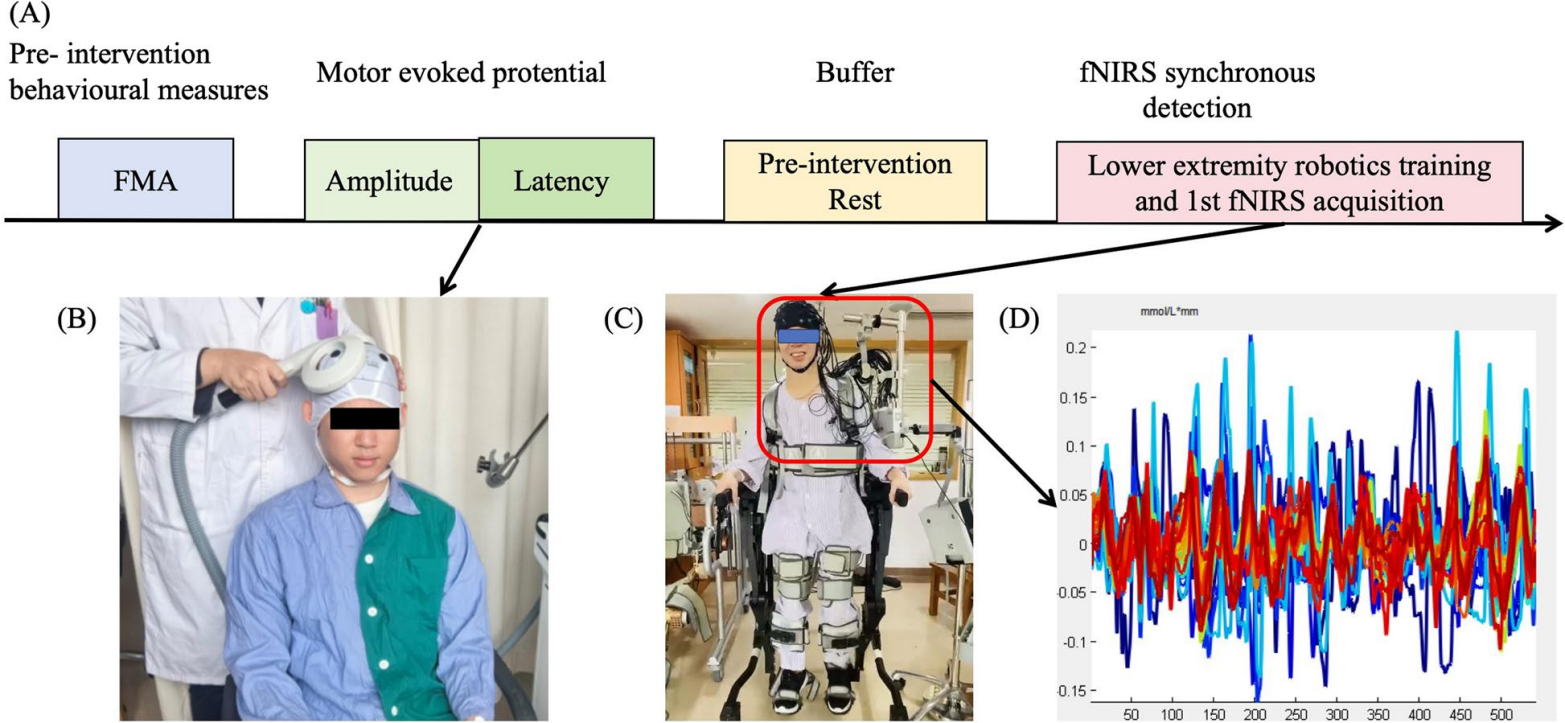
The experimental setup. fNIRS technology was used to detect the real-time hemodynamic signals of stroke patients during lower limb robot intervention. **A** Overall experimental process; **B** MEP acquisition; **C** First completed lower extremity robotics training; **D** Collecting fNIRS data while completing training

The main objective of this study is to evaluate the effect of different lower limb training modes on the recovery of walking function in post-stroke patients. All evaluation indicators will be assessed before the start of treatment (Fig. 1), and two weeks after treatment. For this purpose, the following evaluation indexes will be used:

1. Baseline data collection

Prior to the first intervention, baseline data will be collected from the patients including: age, gender, height, weight, blood pressure, habitual hand use, education level, type of stroke (ischemic or hemorrhagic), date of onset, and cause of onset. Patients with cognitive impairment will also be excluded using cognitive function assessments such as MMSE (Mini-Mental State Examination) [36].

2) Behavioral assessment

Fugl-Meyer Assessment (FMA) was established in 1975 to evaluate the recovery of sensorimotor function in stroke patients [37]. The FMA-Lower Extremity (FMA-LE) assesses movements of the hip, knee, and ankle, recording the hierarchical recovery from reflexive to synergistic and non-synergistic movements based on the Brunnstrom recovery stages. The FMA-LE motor domain employs a 3-point ordinal scale as follows: 0 for unable to perform, 1 for partial performance, and 2 for complete performance, with a possible score range from 0 to 34. Additionally, the assessment evaluates patient coordination, sensory function, joint range of motion, and joint pain.

3) Assessing the motor pathways

The latency and amplitude of MEP of the tibialis anterior muscle elicited by TMS were used to indicate corticomotor excitability. The MEP of the bilateral tibialis anterior muscles were recorded by an electromyographic machine in response to TMS (Magstim 200 electromagnetic stimulation) delivered through a figure-of-eight coil placed on the contralateral motor cortex [38, 39] (Fig. 1 B).

During measurements, participants lay comfortably on a reclined chair with hips and knees in a slightly flexed position and wore a fitted cap marked with a coordinate system (distance, 1 cm). They were instructed to completely relax their lower limbs during the evaluation period. Muscle activity was carefully monitored by real-time electromyography to confirm a relaxed state. The coil was positioned parallel with the coronal plane, and the handle of the coil pointed outward to induce a posterior-to-anterior current flow in the brain. The optimal scalp location (hot spot) was determined by moving the TMS stimulator over the scalp in 1-cm steps. Once the hot spot was identified, a single-pulse TMS was delivered to the location to determine the resting motor threshold, defined as the lowest stimulus intensity necessary to elicit MEP greater than 0.05-mV peak-to-peak amplitude in at least 5 of 10 consecutive stimuli. Single magnetic stimulations at 110% of the resting motor threshold were randomly administered over the motor hot spot of both hemispheres to determine the MEP latency and amplitude for tibialis anterior muscles. In all, 10 sweeps of data were collected with a ≥ 5-s stimulus interval.

4) Measuring hemodynamic responses

Functional near-infrared spectroscopy (fNIRS) was used to assess cortical activation before and after the intervention. A task-based block design was employed, in which patients performed active-assisted gait training during the fNIRS acquisition. Each fNIRS session consisted of alternating blocks of 90 s of walking and 30 s of rest, repeated 5 times. The assessment was conducted while patients were undergoing robot-assisted walking, and the walking mode was consistent with their assigned intervention group—active-assisted mode for the Active-assisted therapy group and passive mode for the Passive therapy group. The Control group completed the same fNIRS task but without any robotic assistance, performing walking task on the robot. This design ensured that all groups participated in similar types of movement tasks during the assessment, while varying the level of assistance provided.

**Fig. 2:**
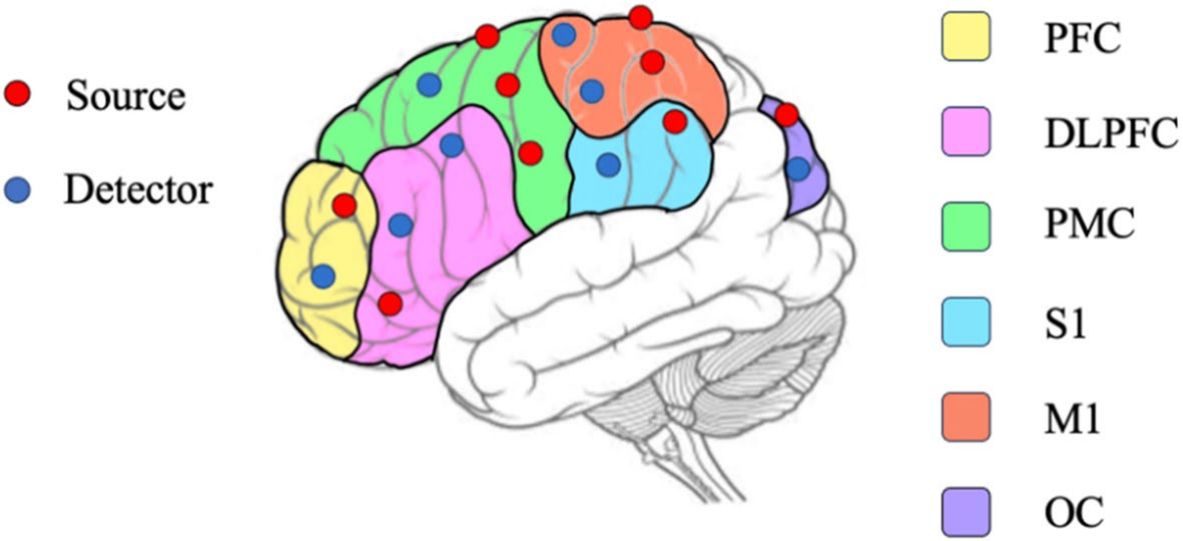
The functional near infrared spectroscopy (fNIRS) optode arrangement. Positioning of the light sources (red) and detectors (blue). The measurement cortex areas cover dorsolateral prefrontal cortex (DLPFC), prefrontal cortex (PFC), premotor cortex (PMC), primary sensory cortex (S1), primary motor cortex (M1), Occipital lobe (OC)

Hemodynamic measurements were performed using a dual-wavelength (740 nm and 850 nm) multichannel continuous wave tissue oxygenation monitor (NirSmart, Danyang Huichuang Medical Devices Co. Ltd., Beijing, China) [40, 41] during the patient's first and tenth lower extremity robotic sessions (Fig. 1 C and D). 38 measurement channels, consisting of 18 light source probes and 16 detector probes mounted on the head, were established according to the International 10–20 system. The channel length was set to 30 mm with symmetrical coverage of ipsilateral and contralateral prefrontal cortex (iPFC/cPFC), ipsilateral and contralateral dorsolateral prefrontal cortex (iDLPFC/cDLPFC), ipsilateral and contralateral premotor cortex (iPMC/cPMC), ipsilateral and contralateral primary motor cortex (iM1/cM1), ipsilateral and contralateral primary somatosensory cortex (iS1/cS1) and ipsilateral and contralateral occipital cortex (iOC/cOC) (Fig. 2). The sampling frequency was kept at 10 Hz [42, 43].

### Statistical analysis

The Kolmogorov–Smirnov test and Levene's test were used to test for normality of variance and homogeneity of data results. To test for normality of variance and homogeneity of data results across groups, within-group comparisons were made using repeated measures ANOVA to assess the significance of the change in assessment scores before and after each group, with significance set at *P* < 0.05. For between-group comparisons, a one-way ANOVA was used to test for differences between groups, and between-group multiple comparisons were made using Bonferroni's correction, with significance set at *P* < 0.05.

## Result

### Demographic characteristic

**Fig. 3:**
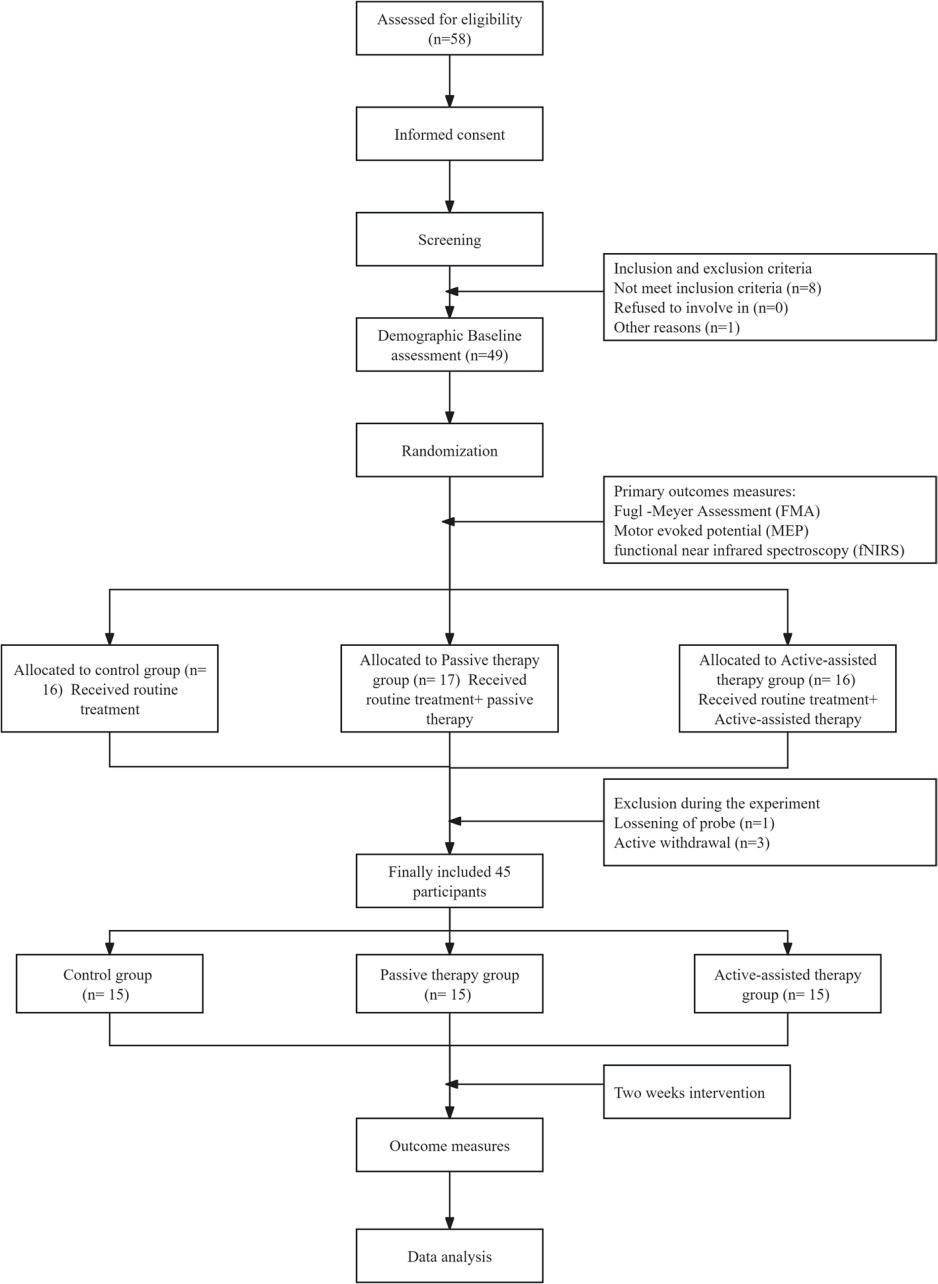
Study flow

The participant flow chart is shown in Fig. 3. A total of 58 inpatients from the Third Affiliated Hospital of Sun Yat-sen University were screened for eligibility, and 45 patients were finally included. The baseline demographic characteristics of the participants are shown in Table 1. There were no significant differences between the groups in terms of age, gender, stroke type, affected side, and time of onset. In addition, there were no significant differences between the three groups in terms of baseline clinical outcomes and brain function.

**Table 1:** Characteristics of patients

		Control group	Passive therapy group	Active-assisted therapy group	*P* value
Genders	Male	10	9	12	0.643
	Female	5	6	3	
Age		52.18 ± 14.41	64 ± 9.36	59 ± 9.21	0.133
Stroke type	Hemorrhage	7	10	5	0.536
	Infarction	8	5	9	
Hemiplegic side	Left	9	7	9	0.808
	Right	6	8	6	
Duration (days)		134.75 ± 131.09	98.3 ± 115.02	101.17 ± 53.32	0.667

### Clinical outcomes

#### Effects on motor function

The main result of this study was that FMA scores and MEP improved significantly over time in all three groups. Among them, the FMA scores of the passive and assisted groups were significantly higher after treatment than before treatment (*P* < 0.01). In addition, the improvement in the active-assisted group was significantly higher than that in the control group (*P* = 0.03), as shown in Table 2.

**Table 2:** Comparison of clinical outcomes between the control, passive and assisted groups

Variable/Group	P_1 VS2_ (Control vs Passive)	P_1 VS3_ (Control vs Active-Assisted)	P_2 VS3_ (Passive vs Active-Assisted)
FMA			
Before- intervention	0.63	0.76	0.85
After- intervention	0.25	0.03*	0.37
MEP			
Before- intervention	0.21	0.74	0.07
After- intervention	0.19	0.27	< 0.01*

P_1 VS2_ = Control group VS Passive therapy group; P_1 VS3_ = Control group VS Active-assisted therapy group; P_1 VS3_ = Passive therapy group VS Active-assisted therapy group.

*means that the comparison is statistically significant

T Post-treatment analysis revealed a significant reduction in MEP peak amplitudes across all groups (*P* < 0.05), indicating enhanced corticomotor excitability following the interventions. Moreover, the assisted group showed a decrease of 8.34 in MEP scores, which was significantly higher than the corresponding decrease in the passive group (Table 3).

**Table 3:** Comparison of clinical outcomes in the control, passive and assisted groups

Variable/Group	Before- intervention	After- intervention	t or z value	*p*-Value
FMA				
Control group	19.40 ± 5.26	19.87 ± 5.59	−0.27	0.80
Passive therapy group	19.58 ± 9.01	23.41 ± 8.07	−4.82	< 0.01^*^
Active-assisted therapy group	19.00 ± 5.27	25.83 ± 7.46	−3.00	0.01^*^
MEP				
Control group	25.20 ± 7.69	22.40 ± 7.35	3.85	< 0.01^*^
Passive therapy group	31.92 ± 9.29	28.59 ± 8.99	2.34	0.04^*^
Active-assisted therapy group	27.42 ± 10.77	19.08 ± 4.23	3.07	0.01^*^

*means that the comparison is statistically significant

**Fig. 4:**
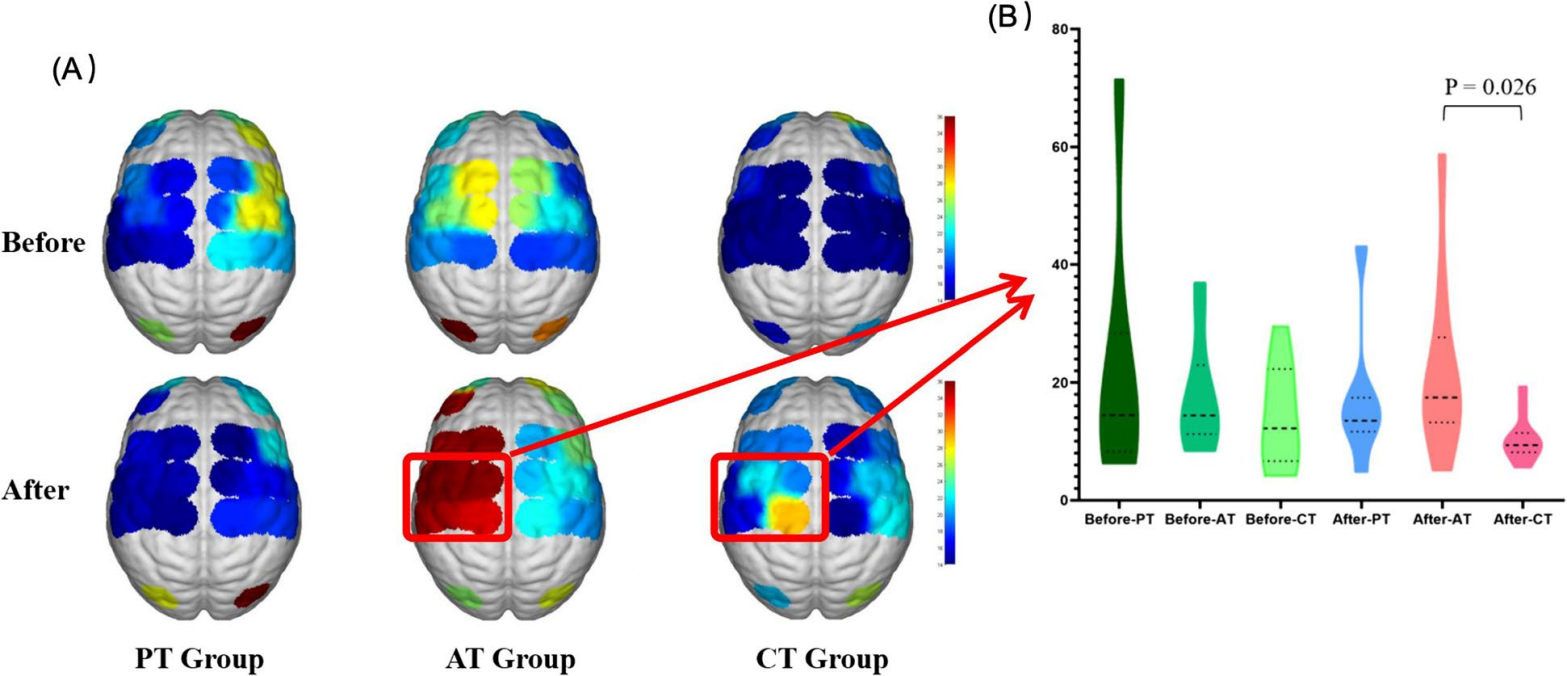
Comparison of brain activation in Control, Passive therapy and Active-assisted therapy groups before and after treatment. PT group = Passive therapy group; AT Group = Active-assisted therapy group; CT Group = control group

Analysis of the pre- and post-treatment fNIRS data for the three groups revealed a more pronounced enhancement of brain activation in the patients in the Active-assisted therapy group. The dorsolateral frontal lobe of the ipsilateral side (*F* = 9.25, *P* = 0.007) was significantly activated in the pre- and post-treatment comparisons of the Active-assisted therapy group. This was not statistically significant in the before-and-after comparisons of the other two groups (Fig. 4A).

For pre-treatment brain activation, there was no significant difference between the three groups. However, after treatment, the between-group comparison of the three groups showed statistical significance in the Active-assisted therapy group. In the post-treatment brain activation comparison between the Active-assisted therapy group and control group, the ipsilateral M1 region (*F* = 5.82, *P* = 0.026) was significantly activated in the assisted group (Fig. 4B). However, the comparisons in the passive group vs the control group and the assisted group vs the passive group were not statistically significant.

## Discussion

The aim of this study was to compare the effects of assisted-mode and passive-mode lower limb robotic training on the recovery of walking function in stroke patients. The results showed that although both training modes significantly improved patients'walking function, Active-assisted therapy group was superior to the Passive therapy group in terms of improvement in motor function and neurophysiological indexes. These findings suggest that active robotic training not only facilitates the recovery of lower limb motor function but also supports neuroplasticity and the reorganization of brain function, thereby offering a dual benefit in stroke rehabilitation [10, 32].

To assess the efficacy on walking function and neuroplasticity, we selected three outcome indicators:

1. FMA: This is a validated clinical scale for assessing motor recovery in stroke patients, particularly focusing on lower limb movement, coordination, and sensory integration, which are directly related to walking function.

From the results of motor function assessment, the FMA scores of both the Active-assisted therapy group and Passive therapy group were significantly higher after treatment than before treatment (*P* < 0.01), which was consistent with the results of previous studies [11, 16]. However, the elevation in the active assisted mode group was significantly higher than that in the passive mode group (*P* = 0.02), suggesting that the assisted mode is more conducive to the recovery of lower limb motor function [9, 15]. This may be due to the fact that the assisted mode can provide appropriate assistive forces to help patients accomplish more voluntary movements and promote the recovery of neuroplasticity [44]. On the contrary, the passive mode, although providing high repetition and consistency of training, lacks the involvement of voluntary movement and may limit neuroplasticity [3, 45].

2) MEP: MEP measures the excitability of the corticospinal tract through transcranial magnetic stimulation (TMS). It serves as an objective neurophysiological marker of motor pathway integrity and plasticity.

In terms of neurophysiological assessment, a significant decrease in peak MEP was observed in both groups after treatment (*P* < 0.05), showing an increase in brain excitability [46]. This result is consistent with previous studies showing that lower limb robotics training promotes cortical control of lower limb muscles [47–50]. More importantly, the assisted-mode group showed significantly higher improvement in MEP scores than the passive-mode group (*P* < 0.01), further supporting the advantage of assisted-mode in promoting neurological recovery [51, 52].

3) fNIRS: fNIRS detects hemodynamic responses in cortical regions such as M1 and S1 during gait-related tasks. This allows assessment of brain activation patterns associated with walking and neuroplastic changes.

The results of near-infrared brain imaging assessment showed that the brain activation in the assisted-mode group was significantly higher than that in the other two groups after treatment, especially on the activation in the M1 area of the affected side (*F* = 5.82, *P* = 0.026). This finding suggests that the assisted movement mode not only contributes to the recovery of lower limb motor function, but also promotes the reorganization and recovery of brain function [53]. In contrast, the relatively weak effect of passive mode on brain activation may be attributed to the lack of active patient participation in passive training, resulting in a limited effect on brain function reorganization [17].

From a theoretical perspective, the superiority of the active-assisted robot training over passive training can be attributed to several key factors related to neuroplasticity, motor control, and sensory-motor feedback.

1. Neuroplasticity and Motor Pathway Reorganization: Active-assisted training requires voluntary participation, which engages the sensorimotor cortex and promotes neuroplasticity. By involving the brain in motor planning and execution, this training helps reorganize neural circuits, particularly in areas responsible for lower limb movement. In contrast, passive training involves minimal brain activation, as patients do not actively control their movements, limiting the brain’s ability to remodel and reorganize motor pathways [54].
2. Voluntary Motor Intent and Execution: In the active-assisted mode, patients are actively involved in the movement process, albeit with some robotic assistance. This participation in movement execution improves motor intention and enhances voluntary control over the affected limbs. Passive training, on the other hand, does not engage motor intention, as the device controls the movement, leaving the brain with little opportunity to strengthen voluntary motor pathways [55].
3. Sensory-Motor Feedback: Active-assisted training provides richer sensory-motor feedback, such as proprioceptive and visual cues, that helps patients adjust their movements and develop better motor coordination. This feedback is crucial for the brain to form and reinforce new motor patterns. Passive training, however, limits sensory feedback since patients are not actively engaged in movement control, thus reducing the brain's ability to optimize motor function [56].

The strengths of this study include the use of a randomized controlled design, strict control of the intervening variables, and a comprehensive assessment of the training effect through multiple evaluation indicators. However, this study also has some limitations. First, the sample size was small, which may affect the generalizability of the results. Second, the study only assessed short-term effects and lacked observation of long-term effects. Therefore, future studies should expand the sample size and conduct long-term follow-up to further validate the effects of different training modes. Third, although all participants received standard rehabilitation care, the control group received fewer training sessions compared to the intervention groups, which might have contributed to differences in outcomes. Future studies should consider equalizing the therapy duration across groups to isolate the specific effects of the intervention type.

## Conclusion

In conclusion, this study suggests that active-mode lower limb robotic training may offer greater benefits than passive-mode training in improving walking function and promoting neurological recovery in stroke patients. Although the sample size was limited, the observed improvements in motor and neurophysiological outcomes indicate that the active-assisted mode has potential as a valuable approach in post-stroke rehabilitation. However, considering the limited number of participants and the relatively high cost of robotic devices, these findings should be interpreted with caution. Further large-scale studies are needed to validate these results and to assess the cost-effectiveness of active robotic rehabilitation before making strong clinical recommendations.

## Data Availability

Data is provided within the manuscript or supplementary information files.
